# Defy Dementia: Mobilizing a Public Health Awareness Campaign for Dementia Prevention

**DOI:** 10.1002/alz.089811

**Published:** 2025-01-09

**Authors:** Allison B. Sekuler, Rosanne Aleong, Shusmita Rashid, Faith Boutcher, Nicole D. Anderson, Sylvain Dubroqua, Natalie Leventhal

**Affiliations:** ^1^ McMaster University, Hamilton, ON Canada; ^2^ Rotman Research Institute, Toronto, ON Canada; ^3^ University of Toronto, Toronto, ON Canada; ^4^ Baycrest Academy for Research and Education, Toronto, ON Canada; ^5^ Centre for Aging + Brain Health Innovation, Toronto, ON Canada; ^6^ Kimel Family Centre for Brain Health and Wellness, Toronto, ON Canada; ^7^ Ben & Hilda Katz Interprofessional Research Centre in Geriatric and Dementia Care, Toronto, ON Canada

## Abstract

**Background:**

While age is the most significant risk factor for dementia, increased awareness and understanding of other modifiable risk factors of dementia, coupled with proactive lifestyle behavior changes, hold the potential to prevent dementia and improve the quality of life for older adults.

Defy Dementia is a public health initiative, led by the Baycrest Academy for Research and Education (BARE) and funded by the Public Health Agency of Canada. It involves curating, co‐designing, and disseminating a series of knowledge products to raise public awareness of dementia prevention and reduce stigma associated with dementia. These knowledge products, including podcasts, minute‐videos, and infographics, aim to raise awareness about modifiable risk factors associated with dementia and empower individuals to take proactive measures.

**Method:**

To assess the impact of our knowledge products our evaluation consisted of two main components: a) quantitative data obtained through an online survey on the project website (convenience sample); and b) qualitative data obtained through 4 focus groups (each with a selected group of 4‐8 participants, derived from the first convenience sample and event participants). Quantitative data will be exported from REDCap and explored through descriptive statistics using SAS System version 9.4 or R version 4.2. Thematic analysis of the focus group data will be performed using NVivo R.

**Result:**

Based on our hypothesis, we anticipate that individuals who engage with the knowledge products or attend an event will experience an improvement in their awareness and understanding of the modifiable risk factors associated with dementia, have enhanced knowledge about dementia prevention, exhibit behaviour change and have changed attitudes about PLWD.

**Conclusion:**

We will demonstrate how meaningfully engaging older adults, persons living with dementia, and their care partners in the design and dissemination of knowledge can not only improve the relevance and uptake of the knowledge but also foster empathy and reduce stigma associated with dementia. We will also engage the audience to consider how increased awareness of modifiable risk factors for dementia, coupled with action, has the potential to lower the risk of developing dementia, ultimately leading to its prevention or delay and enhancing the quality of life for older adults.

